# A Manual of Diseases of the Nervous System

**Published:** 1899-12

**Authors:** 


**A Manual of Diseases of the Nervous System.** By Sir W. R. Gowers, M.D., F.R.S. Third Edition. Vol. I.—*Diseases of the Nerves and Spinal Cord.* Pp. xvi., 692. London: J. & A. Churchill. 1899.

Sir W. R. Gowers's *Manual of Diseases of the Nervous System* is so well known and widely appreciated, that this third edition of his first volume will be read with much interest. At the same time in the case of a book that is so well recognised as a standard and trustworthy authority on the subject and as a model of clear exposition it is superfluous to do more than indicate the differences from the previous editions. Dr. James Taylor is associated with Sir W. R. Gowers as joint-editor of this volume. As was inevitable the book is enlarged, and is well abreast of the advances made in the neurology of the nerves and spinal cord since the last edition. A new chapter is added which gives an excellent summary of the changes made during the last few years in our conceptions of the constitution of the

Nervous System and their bearing on its physiology and pathology. The section on Neuritis has been extended; and there are separate sections on Beri-beri and Herpetic Neuritis. In tabes dorsalis a positive history of syphilis or of a hard chancre was obtained in 77 per cent. of private patients, and it is stated that in less than 10 per cent. can syphilis be excluded with confidence. There is a good account of sclerosis of the cord from toxic blood states, which includes Pellagra, and is illustrated by some excellent figures. The account of the muscular dystrophies and idiopathic muscular atrophies is very full and good; the peroneal form of amyotrophy, with its tendency to affect more than one member of a family, has for a sub-title "Neuritic Muscular Atrophy," and the evidence for regarding the disease as due to a "peripheral neuritis, motor or total, of extremely chronic course and peculiar origin" is discussed.

Enough has been said to show that the book, whilst retaining those features which have made it such an admirable guide to practitioners and students in the past, contains in this new edition much added matter of value. We must not omit to mention the appendix dealing with the muscle spindles.

